# IL-33/ST2 Pathway and Galectin-3 as a New Analytes in Pathogenesis and Cardiometabolic Risk Evaluation in Psychosis

**DOI:** 10.3389/fpsyt.2018.00271

**Published:** 2018-06-22

**Authors:** Milica M. Borovcanin, Slavica M. Janicijevic, Ivan P. Jovanovic, Nevena Gajovic, Nebojsa N. Arsenijevic, Miodrag L. Lukic

**Affiliations:** ^1^Department of Psychiatry, Faculty of Medical Sciences, University of Kragujevac, Kragujevac, Serbia; ^2^Faculty of Medical Sciences, Center for Molecular Medicine and Stem Cell Research, University of Kragujevac, Kragujevac, Serbia

**Keywords:** schizophrenia, galectin-3, interleukin-33, metabolic syndrome, cardiovascular issues

## Abstract

Schizophrenia and treatment of this disorder are often accompanied with metabolic syndrome and cardiovascular issues. Alterations in the serum level of innate immune mediators, such as interleukin-33 (IL-33) and its receptor IL-33R (ST2) and Galectin-3 (Gal-3) were observed in these conditions. Moreover, these parameters are potential prognostic and therapeutic markers. There is also accumulating evidence that these molecules play a role in neuroinflammation. Therefore, in this study we have investigated the serum level of Gal-3, IL-33 and soluble ST2 (sST2) in different stages of schizophrenia. Gal-3 levels were elevated in remission and lower in schizophrenia exacerbation in comparison with controls. Levels of IL-33 and sST2 are higher in schizophrenia exacerbation in comparison with controls and patients in remission. This initial analysis of new markers of neuroinflammation suggested their involvement in schizophrenia pathophysiology and/or cardiometabolic comorbidity.

## Introduction

The novel therapeutical strategies have encountered new problems in treatment of patients with schizophrenia (1). Although efficient in resolving the positive symptoms and mitigating the extrapyramidal sympthomatology, the use of atypical antipsychotics in schizophrenia was linked to higher prevalence of patients with metabolic syndrome (2) and cardiovascular issues (3, 4). Diagnostic and treatment algorithms incorporated predictive values of Galectin-3 (Gal-3) and interleukin-33 (IL-33) in treatment of coronary diseases and heart failure (5, 6) and evaluating prediabetic state (7). The dogma about immune privilege of the brain is now revisited again (8) and this bidirectional communication implicated the usefulness of peripheral markers and indicated that underlying mechanisms of somatic states frequently observed in schizophrenia should be explored further.

Galectins present animal lectins family, that have the affinity for β-galactosides and could interact with cell-surface and extracellular matrix glycoproteins through lectin-carbohydrate interactions (9). The most studied and unique family member is Gal-3 (10). Gal-3 is found in different cell and tissue types, and its various functions have been described, like promotion of cell migration, stimulating role in proliferation, differentiation, survival, adhesion, apoptosis, and immune responses (11). Gal-3 can be expressed in cytoplasm, nucleus, mitochondria, and cell surface, and it can also be secreted by macrophages and monocytes and other various cell types into the extracellular matrix and circulation (12, 13). Intracellular Gal-3 can be transported to the cell surface or even secreted outside of cells and depending on localization Gal-3 could act as positive or negative regulator of apoptosis [reviewed in (14)]. We postulated that extracellular Gal-3 is most important in interaction that leads to inflammation, as shown in lipopolysaccharide induced neuroinflammation (15). This can be only formally proven by using different inhibitors of Gal-3 in experimental models. Deletion of the Gal-3 gene has influence on deterioration of diabetes mellitus (16) and could predict vascular complications in patients with type 2 diabetes mellitus (17). In obesity in animal models and in humans, elevated serum levels of Gal-3 seem to have protective function (18). Gal-3 showed to be included into myocardial fibrosis and remodeling (19) and circulating Gal-3 was associated with cardiometabolic disease in the community (20).

Experimental studies have shown that Gal-3 gene expression is upregulated after neuronal damage (21), in traumatic spinal cord injury (22) and in experimental autoimmune encephalomyelitis (23). Also, it is expressed in activated adult microglial cells in the ischemic lesion and it is required for resident microglia activation and proliferation (24). Gal-3- deficient mice showed to be protected against ischemic injury, particularly in the hippocampus and striatum (21). Considering Gal-3 regulating properties especially in the hippocampus, Trompet et al. (25) hypothesized that Gal-3 may also play a role in cognitive functioning. Elevated Gal-3 sera levels were measured in patients with Alzheimer's disease and Mini-Mental Status Examination score, as a measure for cognitive status, was found to correlate with the Gal-3 serum levels in both, in the patients and healthy controls (26).

IL-33, as an IL-1 family member, has a role in initiation of inflammation, its regulation and maintenance (27). IL-33 is a ligand for receptor complex of two proteins, binds to ST2 and forms suitable conformation to contact with IL-1 receptor accessory protein. ST2 has two forms: trans-membranes full-length (ST2L) and soluble form (sST2), which binds directly to IL-33 and has a role as a decoy receptor to competing with membrane bound ST2 (28). It seems that after secreted into the circulation, sST2 inhibits the effects of IL-33/ST2L signaling and attenuates the systemic effects of IL-33 (29). IL-33/ST2 signals have anti- or pro- inflammatory effects in diseases by activating cells of innate and adaptive immune systems, but it is still unclear what kind of immune cells are first induced to produce IL-33 (28). IL-33 can have protective properties in atherosclerosis development (30) and blood concentrations of sST2 are markedly increased in cardiac diseases (31) and metabolic syndrome (32).

During the brain development both precursors of astrocytes and oligodendrocytes express IL-33 and its detection during first postnatal week coincides with very important neurodevelopmental phases, suggesting a role of IL-33 in the absence of an inflammatory response (33). Genetic study showed decreased IL-33 expression in the brain of Alzheimer‘s disease patients (34). IL-33 polymorphism was associated with risk for schizophrenia (35) and recently de Campos-Carli et al. (36) have measured similar sera concentrations of IL-33 and sST2 in patients with chronic schizophrenia and established significant correlation between levels of these cytokines and cognition in chronic schizophrenia.

Consequently, we wanted to investigate the alterations of innate inflammatory markers Gal-3, IL-33, and sST2 in different stages of schizophrenia and to explore the possible correlation of their serum concentrations with clinical symptomatology and laboratory parameter.

## Experimental procedures

### Participants

Subjects included in this study were: drug naïve patients with First Episode Psychosis-FEP (*n* = 77); patients with Schizophrenia in relapse—SC in relapse (*n* = 45) previously treated with antipsychotics; patients with Schizophrenia in remission—SC in remission (*n* = 27); and healthy control—HC subjects (*n* = 18). The patients with FEP and SC in relapse were recruited during the previous project [data published in (37–39)] and patients with SC in remission were enrolled during 2016 at Psychiatric Clinic, Clinical Centre Kragujevac, after a 3 month stable depot antipsychotic therapy of risperidone or paliperidone. Healthy control subjects were recruited at Service Supply of Blood and Blood Products, Clinical Centre Kragujevac. Studies were approved by the Ethic Committee and were conducted in compliance with the ethical principles of the Declaration of Helsinki. Patients were informed and written consent was obtained from all of the patients before starting any study procedure.

Diagnoses were established using International Statistical Classification of Diseases and Related Health Problems, Tenth Revision (ICD-10) (40) criteria for acute psychotic episode (F23) and schizophrenia (F20). Complete medical history was obtained; physical examination and laboratory testing were done. The exclusion criteria considered any severe somatic comorbidity, especially current infections, autoimmune disorders, metabolic disorders, or current anti-inflammatory or antiviral medications. Neither the psychotic patients nor controls have previously suffered from substance or alcohol abuse, nor were other mental illnesses diagnosed as dual diagnoses.

### Psychological assessment

Psychopathology was evaluated using the Positive and Negative Syndrome Scale of Schizophrenia (PANSS) consistent of positive, negative and general psychopathology subscale (41). Criteria for the diagnosis of schizophrenia in remission were lower scores (three or less) on eight diagnostically relevant symptoms in the PANSS: P1, G9, P3, P2, G5, N1, N4, and N6 (42).

### Blood collection and cytokine measurements

The blood samples were collected in the morning (~8 a.m.) and participants were fasting before sampling. Blood clot was cut, than centrifuged and after separation the serum samples were stored at −20°. Serum levels of Gal-3, IL-33, and sST2 were measured using sensitive Enzyme-Linked ImmunoSorbent Assay (ELISA) kits specific for the human cytokines, following the instructions of the manufacturer (R&D System, Minneapolis, MB). The procedure has been described in detail previously (37–39) and performed at the Center for Molecular Medicine and Stem Cell Research, Faculty of Medical Sciences, University of Kragujevac.

### Statistical analysis

The data were presented as means, standard deviation (SD), standard errors (SE), and median. The distribution of data was tested with Shapiro-Wilk test and further statistical analysis was performed using parametric and non-parametric tests. Mann-Whitney test was used to evaluate the significance of differences of parameters between two examined groups. Kruskal-Wallis test was used to examine the difference of parameters among groups. The possible relationships between patients‘ serum cytokine levels and clinical scores were evaluated using the Pearson's correlation and between laboratory parameters and clinical scores using the Spearman's correlation. A *p*-value of 0.05 was considered to be statistically significant. The statistical analyses were performed using SPSS 20.0 software.

## Results

### Demographical and clinical data

The control group consisted of 18 healthy subjects (6 men vs. 12 women), with mean age of 37.67 ± 9.96 and without significant age difference in comparison with patients' groups. Table 1 presents demographic and clinical characteristics of the patients. Significant difference was observed in duration of illness among groups of patients, showed in Table 1 (FEP vs. SC in relapse vs. SC in remission: 0.28 ± 1.93 vs. 7.31 ± 6.30 vs. 9.95 ± 7.71 years; *p* = 0.000), with no difference in gender distribution comparing with control group. Comparison of PANSS scores and subscores reveals differences in positive, negative and general psychopathology scores between patients. Mean value of negative subscores was higher in SC in relapse, than those in FEP patients (FEP vs. SC in relapse: 21.75 ± 5.90 vs. 26.20 ± 9.98; *p* = 0.006). Patients with SC in remission have significantly lower positive and general subscores (*p* = 0.007 and *p* = 0.004, respectively), with higher negative subscores (*p* = 0.000) than patients with FEP. Differences in PANSS subscores were established in lower positive (*p* = 0.007) and lower general subscores (*p* = 0.001) in patients with SC in remission compared with SC patients in relapse.

**Table 1:** Demographic data and clinical disability measures.

**Parameter**	**Gender Men/women**		**Age(Years; mean ± SD)**	**Duration of illness (Years; mean ± SD)**	**PANSS positive score**	**PANSS negative score**	**PANSS general score**	**PANSS total score**
FEP	36	52	33.64 ± 8.84	0.28 ± 1.93	25.73 ± 5.99	21.75 ± 5.90	53.56 ± 7.27	101.03 ± 14.62
SC in relapse	17	28	35.95 ± 11.40	7.31 ± 6.30	26.53 ± 6.29	26.20 ± 9.98^*^	56.44 ± 12.67	105.86 ± 19.79
SC in remission	11	16	36.19 ± 9.28	9.95 ± 7.71	22.26 ± 5.97^**^^,^^***^	27.52 ± 6.10^**^	9.44 ± 7.83^**^^,^^***^	99.22 ± 18.24

* *Mann-Whitney test, statistically significant difference between FEP and SC in relapse groups (p = 0.006)*.

** *Mann-Whitney test, statistically significant difference between FEP and SC in remission groups (p < 0.05)*.

*** *Mann-Whitney test, statistically significant difference between SC in relapse and SC in remission groups (p < 0.05)*.

Patients with SC in remission were treated with depot formulation of atypical antipsychotics risperidone in a dose range of 25–50 mg (∑*n* = 22) and paliperidone in a dose range of 75–150 mg (∑*n* = 5). Laboratory analysis parameters are presented in Table 2.

**Table 2:** Laboratory values of cardiometabolic parameters.

**Parameter**	**Fasting glucose (mMol/L)**	**Total cholesterol (mMol/L)**	**Triglycerides (mMol/L)**	**HDL (mMol/L)**	**LDL (mMol/L)**	**CK (IU/L)**	**CK-MB (IU/L)**
FEP	5.04 ± 1.38	5.17 ± 2.97	3.60 ± 2.15	1.35 ± 0.42	2.89 ± 1.21	437.46 ± 1275.16	ND^a^
SC in relapse	5.01 ± 2.10	4.86 ± 1.37	1.32 ± 1.03	1.33 ± 0.38	3.33 ± 2.35	533.10 ± 1346.93	ND^a^
SC in remission	5.27 ± 2.14	5.90 ± 1.38	1.80 ± 1.31	1.34 ± 0.31	3.66 ± 1.14	119.33 ± 92.82	19.19 ± 4.26

a *ND, not done*.

### Higher serum concentrations of IL-33 and sST2 in exacerbation of early schizophrenia

**Figure 1:**
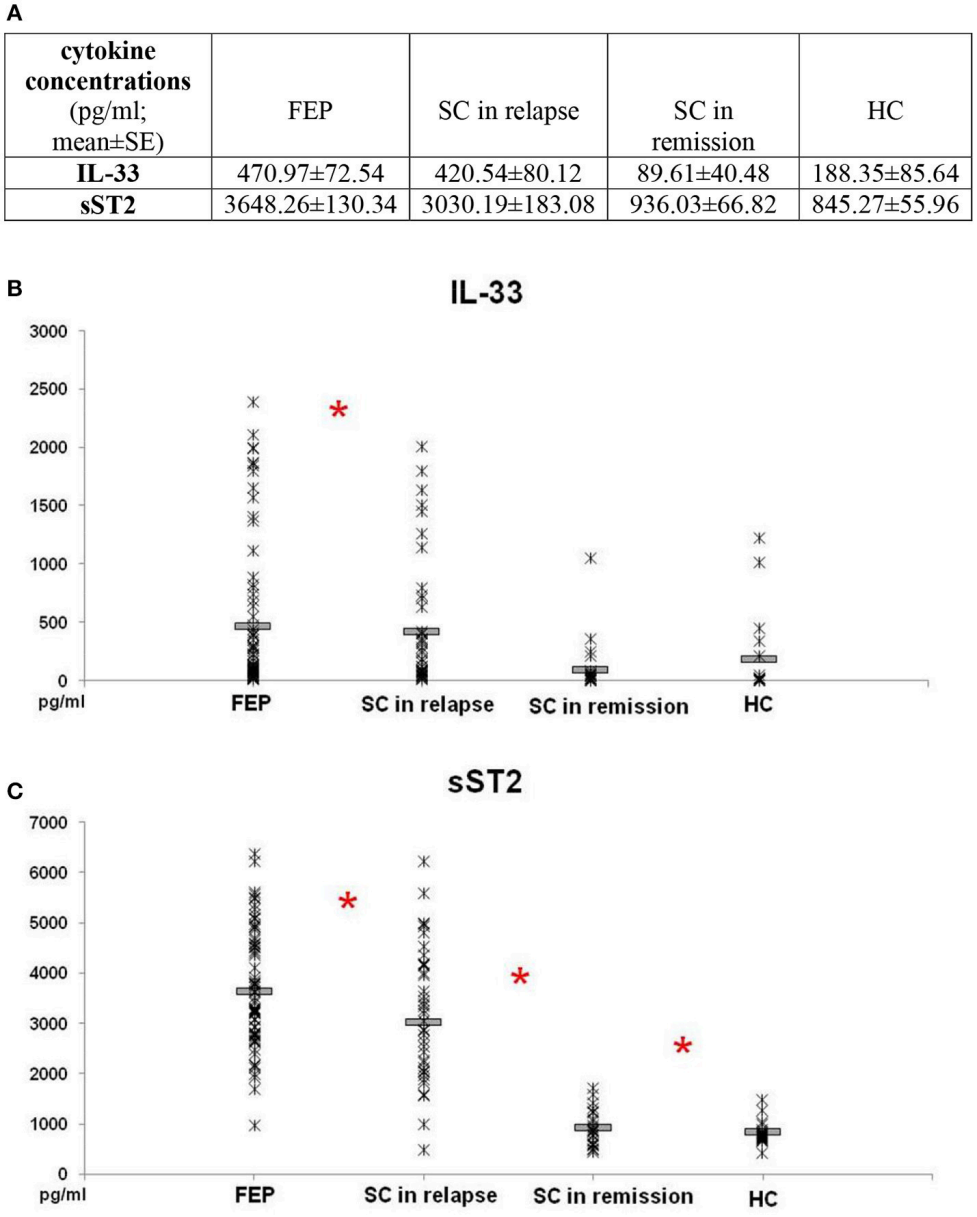
Serum levels of IL-33 and sST2 in FEP patients (*n* = 77), SC in relapse (*n* = 45), SC in remission (*n* = 27) and healthy subjects (*n* = 18) were determined by ELISA. Data presented as mean ± SE in Table **(A)**. Statistical significance was tested by Kruskal-Wallis and Mann-Whitney test (*p < 0.05). Serum concentrations of IL-33 were higher in FEP and SC in relapse, than SC in remission and healthy control subjects (*p* ≤ 0.001) **(B)**. Serum concentrations of sST2 were higher in patients with FEP compared to SC in relapse, than higher values were observed in patients with SC in relapse compared with SC in remission (*p* ≤ 0.01) and SC in remission than control subjects **(C)**.

Comparison of IL-33 serum levels between FEP and SC in relapse group did not reveal statistically significant difference (*p* = 0.869). Also, there was no difference in serum levels of IL-33 between SC in remission and HC subjects (*p* = 0.871). IL-33 sera levels were significantly higher in FEP patients compared to SC in remission and those in HC (FEP vs. SC in remission vs. HC: 470.97 ± 72.54 vs. 89.61 ± 40.48 vs. 188.35 ± 85.64 pg/ml; *p* = 0.000). Comparing serum concentrations of IL-33 in SC in relapse with those in remission and HC also show statistically significant difference (*p* = 0.000 and *p* = 0.001, respectively) (Figure 1).

While conducting the group cross-comparison of serum sST2 levels, the grading in descending manner was observed (Figure 1). The statistically significant higher values of sST2 were measured in patients with FEP compared to SC in relapse (3648.26 ± 130.34 vs. 3030.19 ± 183.08 pg/ml; *p* = 0.010), higher values were observed in patients with SC in relapse compared with SC in remission (3030.19 ± 183.08 vs. 936.03 ± 66.82 pg/ml; *p* = 0.000), with no difference between serum levels of sST2 in patients with SC in remission and healthy control group (936.03 ± 66.82 vs. 845.27 ± 55.96; *p* = 0.391).

### Correlations of IL-33 serum levels with positive and general PANSS scores

**Figure 2:**
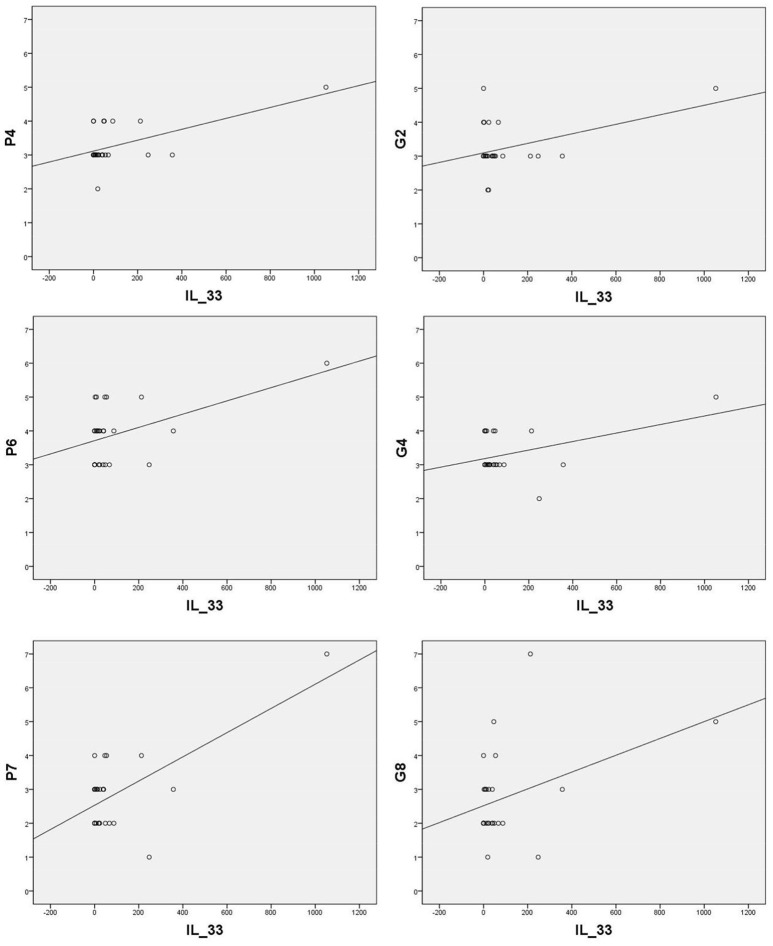
Significant correlation of IL-33 with positive PANSS items [P4 (*r* = 0.570; *p* = 0.002), P6 (*r* = 0.486; *p* = 0.010), P7 (*r* = 0.664; *p* = 0.000)] and general PANSS symptoms [G2 (*r* = 0.424; *p* = 0.028), G4 (*r* = 0.435; *p* = 0.023) and G8 (*r* = 0.396; *p* = 0.041)].

Sera levels of IL-33 in remission are in significant correlation with the PANSS items of positive symptoms [excitement - P4 (*r* = 0.570; *p* = 0.002), suspiciousness/persecution - P6 (*r* = 0.486; *p* = 0.010), and hostility - P7 (*r* = 0.664; *p* = 0.000)] and general symptoms [anxiety - G2 (*r* = 0.424; *p* = 0.028), tension - G4 (*r* = 0.435; *p* = 0.023), and uncooperativeness - G8 (*r* = 0.396; *p* = 0.041)] (presented in Figure 2).

### Serum concentrations of Gal- 3 in patients with schizophrenia are lower in exacerbation and higher in remission compared with healthy subjects

**Figure 3:**
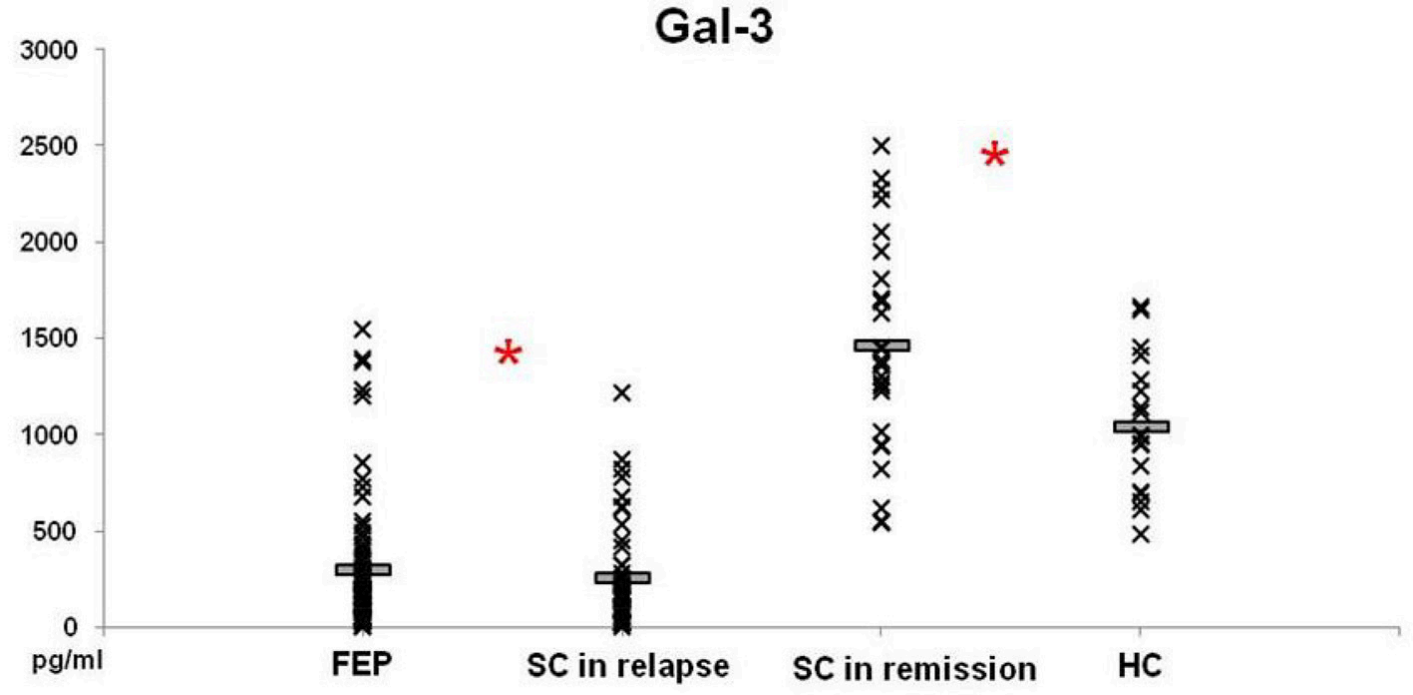
Serum levels of Gal-3 in FEP patients (*n* = 77), SC in relapse (*n* = 45), SC in remission (*n* = 27) and healthy subjects (*n* = 18) were determined by ELISA. Data presented as mean ± SE. Statistical significance was tested by Kruskal-Wallis and Mann-Whitney test (*p < 0.05). Serum concentrations of Gal-3 were significantly lower in FEP and SC in relapse groups and higher in SC in remission, compared with group of healthy control subjects.

Levels of Gal-3 in patients with FEP and SC in relapse were not significantly different between these groups of patients (297.52 ± 37.86 vs. 252.75 ± 41.35 pg/ml; *p* = 0.230), but lower levels were measured in both groups compared with those in HC (*p* = 0.000) (Figure 3). In patients with SC in remission significantly higher levels of Gal-3 were observed in comparison with concentrations measured in patients with FEP (1457.89 ± 104.60 vs. 297.52 ± 37.86 pg/ml; *p* = 0.000), SC in relapse (1457.89 ± 104.60 vs. 252.75 ± 41.35 pg/ml; *p* = 0.000), and HC (1457.89 ± 104.60 vs. 1044.28 ± 83.37 pg/ml; *p* = 0.011). Binary logistic regression analysis revealed that increased levels of Gal-3 influence on the illness onset [Odds Ratio 0.998 (0.996–1.000)]. There was no correlation between Gal-3 sera levels with positive, negative, general and total PANSS scores (data not presented).

### Correlation of serum sST2 with negative scores, cholesterol and cardiac troponin levels

In patients with acute psychosis, sera levels of sST2 were in negative correlation with N subscore (*r* = −0,184; *p* = 0.044) and in patients with SC in remission sera levels were in positive correlation with item P7 (*r* = 0.413; *p* = 0.032), stereotyped thinking - N7 (*r* = 0.384; *p* = 0.048). In patients with schizophrenia in remission negative correlation was observed between serum concentrations of sST2 and levels of cholesterol (*r* = −0.434; *p* = 0.024), Low-Density Lipoprotein (LDL) (*r* = −0.479; *p* = 0.011) and positive correlation with Creatine Kinase - MB (CK-MB) levels (*r* = 0.460; *p* = 0.016).

## Discussion

In this study we evaluated the serum level of two novel participants in innate immunity in patients with psychosis. We have shown that Gal-3 levels were lower in FEP and SC in relapse and higher in SC in remission than those measured in control subjects. We observed higher serum levels of IL-33 and sST2 in patients with FEP and SC in relapse, compared with those in remission and healthy control subjects. In remission, the positive correlation of sera levels of IL-33 was established with positive symptoms of excitement, suspiciousness/persecution, and hostility, but also with general symptoms of anxiety and tension. Serum level of sST2 in acute psychosis negatively correlated with N subscore, but in remission correlated positively with hostility and stereotyped thinking. Measurements in remission reveal the negative correlation of sST2 levels with cholesterol and LDL levels, but positive correlation with cardiac troponin CK-MB was observed.

There is clear importance of Gal-3 in development of nervous system and in neuroinflammation. Gal-3 plays a role in the modulation of immune/inflammatory function, with both pro- and anti-inflammatory actions, depending on multiple factors, such as inflammatory setting and target cell/tissue (11, 43, 44). It is well known that Gal-3 regulation of type-1/type-2 immune response in asthma was presented with lower airway type-2 response in Gal-3^−/−^, but a higher type-1 response compared to Gal-3^+/+^ mice, indicating that Gal-3 facilitates type 2 immune response (45). Also, asthma and schizophrenia cooccurrence was established (46, 47). Kajitani et al. (48) recently reported that the serum Gal-3 levels are elevated in chronic schizophrenia. Thus, it is not surprising to find lower level of Gal-3 in patients with FEP and SC in relapse and higher level in SC in remission (Figure 3) and it is in line with our previous finding of type-2 immune response predominance in these patients (37). We believe that Gal-3 acts as a proinflammatory lectin in patients with schizophrenia. Further, elevation of Gal-3 in chronic schizophrenia could initiate myocardial fibrosis, metabolic changes, and may have protective properties in type-2 diabetes. Gal-3 could be a mediator of underlying mechanisms in schizophrenia onset and cardiovascular and metabolic changes in these patients.

We have shown here that the level of IL-33 is not significantly altered in schizophrenia patients in remission (Figure 1). However, there is clear statistically significant increase in IL-33 levels in patients with exacerbation (Figure 1) and correlation of its levels with positive symptoms scores (Figure 2). Earlier studies have shown different role of IL-33 in inflammatory diseases; immunosupressive role in obesity, atherosclerosis and experimental fulminant hepatitis and proinflammatory role in asthma and antigen-induced arthritis (49–51). Although, IL-33 was initially considered to be a proinflammatory cytokine, its linkage with regulatory T (Treg) immune response was later suggested (51). Recent data have shown that IL-33 downregulates immune response in autoimmune processes (52). It is well known that IL-33 is abundantly present in the central nervous system (CNS) (53). It is particularly highly expressed during early development (33), as well as in inflammatory disease in CNS such as experimental autoimmune encephalitis, an animal model of multiple sclerosis (54). Also, it is reported that IL-33 can modulate microglia in an animal model of Alzheimer‘s disease (55), but its function in these condition is still unclear. It is established that IL-33 acts as alarmin, meaning that this cytokine is released from cells during tissue damage, and not apoptotic cells (56). We assume that during CNS damage, neuroinflammation is followed by IL-33 release from necrotic cells and increment of its serum levels in patients with schizophrenia. It should be added that in atherosclerosis, IL-33 was protective (30). Thus, it is possible that IL-33 production is an attempt to limit inflammation accompanying relapse in schizophrenia. However, direct pathogenic effect cannot be excluded as ST2 dependent Th2 pathology reported to be common denominator in asthma and schizophrenia (57). Considering that sST2 binds directly to IL-33 or acts as a decoy receptor when competing with membrane bound ST2 [reviewed in 28], higher systemic level of sST2 in FEP and SC patients in relapse as well as negative correlation of this molecule with N subscore in acute psychosis may represent compensatory mechanism in suppressing IL-33-dependent inflammation.

The positive correlation of IL-33 with positive PANSS symptoms in remission suggests its potential role in underling mechanisms of psychosis onset. sST2 could have neutralizing properties in the context of excessive IL-33 secretion and also in amelioration of negative symptoms. Although direct correlation of Gal-3 levels with clinical symptoms was not established, some other molecular mechanisms involved in Gal-3-dependent regulation could preserve cognitive potentials in patients with schizophrenia. Considering involvement of Gal-3 and the IL-33/ST2 pathway interactions in the somatic states (58), this interplay could be also involved in onset, clinical presentation and somatic comorbidity of psychosis.

As discussed by Mueller and Dieplinger (59), plasma concentrations of these two analytes have been incorporated in 2013 ACCF/AHA guidelines for additive risk stratification in acute and chronic heart failure (60) and Gal-3, sST2 and BNP were all useful as predictors of 1-year all-cause mortality (6). It was previously presented that life expectancy of patients with schizophrenia is 10–25 years shorter than in general population (61) and 40–50% of premature deaths have been due to cardiovascular diseases (62). Patients with schizophrenia are reported to be three times as likely to experience sudden cardiac death (63). Hou et al. (4) have shown that a history of aggressive behaviors is strongly associated with sudden cardiac death in patients with schizophrenia. Although CK-MB levels were measured additionally only in stable state in our study, in spite of that, the positive correlation with serum sST2 was established. Simultaneously presented elevation of sST2 and higher scores on items equivalent of aggressive behavior suggest that these new inflammatory markers should be considered in additional monitoring of cardiac symptoms that occur without warning in schizophrenia.

Innate immune system primarily initiates defense against pathogens, but also contributes to adaptive induction of sickness behavior and infection recovery (64). In the previous few years central nervous system was no longer viewed as an immunologicaly isolated space, but it seems that its dynamic interaction with the peripheral immune system regulates the activity of immune cells within the central nervous system (65). Childhood traumatic events could have a significant impact by changing the immune response and precipitating further vulnerability for psychiatric disorders and somatic states later in life (64). It is now obvious that metabolic dysregulation in patients with schizophrenia already exists before antipsychotic treatment (66, 67). The fact that schizophrenia risk is driven by genes that not have direct relevance to disease, suggests that schizophrenia could rather be considered as developmental physiological defect (68).

## The limitations of the study

The sample size of the healthy control subjects is rather small compared to other groups (FEP, SC in relapse). The data considering potential confounding factors, such as body mass index, cigarette smoking, and the use of alcohol or other illicit drugs were not collected in patients with FEP and SC in relapse, so it was not possible to include these data into assessment. The possible impact of antipsychotics on cytokine profiles could not be excluded (69), so the further analysis of diverse antipsychotics‘ influences on these specific biomarkers should be done.

## Conclusions

The study of two pathways of innate immunity in schizophrenia revealed that the serum levels of IL-33 and its soluble receptor was unaltered in stable disease, but was significantly enhanced in exacerbation and accompanied with hostility and elevation of cardiac troponin levels. Further, Gal-3 is increased in the serum of schizophrenia patients in remission and seems to be involved in schizophrenia onset. Taken together, this initial analysis of new markers of inflammation suggested their involvement in schizophrenia pathogenesis and cardiometabolic comorbidity.

## Author contributions

All authors equally contributed in the planning, designing and conducting this research. MB and SJ selected the patients, did psychological assessment and statistical analysis and wrote about psychiatric aspects of this topic. IJ, NG, MB, and SJ have done cytokine measurements. MB and IJ designed figures and tables. IJ and NG wrote about immunological underlying mechanisms. NA and ML wrote the introduction and discussion and made integral version of the manuscript. All authors approved the final manuscript.

### Conflict of interest statement

The authors declare that the research was conducted in the absence of any commercial or financial relationships that could be construed as a potential conflict of interest.
